# Translating *in vitro* gut microbiota models to human context: compositional correlations under dietary fiber intervention

**DOI:** 10.3389/fmicb.2025.1708906

**Published:** 2025-12-18

**Authors:** Femke P. M. Hoevenaars, Torsten P. M. Scheithauer, Boukje C. Eveleens Maarse, Isabela M. de Oliveira, Ines Warnke, Wilbert Sybesma, Matthijs Moerland, Tim J. van den Broek, Frank H. J. Schuren

**Affiliations:** 1 Department of Microbiology & Systems Biology, Netherlands Organisation for Applied Scientific Research (TNO), Leiden, Netherlands; 2 Centre for Human Drug Research, Leiden, Netherlands; 3 Leiden University Medical Center, Leiden, Netherlands; 4 DSM Nutritional Products Ltd., Kaiseraugst, Switzerland; 5 Microbiome Solutions, Niederhünigen, Switzerland

**Keywords:** dietary fibers, gastrointestinal health, gut microbiota, *in vitro* systems, *in vivo* translation

## Abstract

**Introduction:**

Large interindividual variation in human gut microbiota composition and its response to interventions limits the development of novel microbiota-targeted supplements. *In vitro* models reflecting this interindividual variation and predicting individual *in vitro* microbiota responses would allow for the assessment of the potential efficacy of such interventions.

**Methods:**

Here, we investigated whether in vitro microbiota modulation by a dietary fiber mixture is translatable to in vivo microbiota outcomes. A 12-week double blind, randomized, placebo-controlled, crossover study with a dietary fiber mixture of acacia gum (AG) and carrot powder was performed in healthy volunteers (*N* = 54, 45–70 years, BMI 27.3 ± 1.4 kg/m^2^). The in vitro platform utilized fecal samples from the same individuals who participated in the in vivo study.

**Results:**

A significant effect on microbiota composition was shown in vivo, although with strong individual variation. The fiber intervention was mimicked in vitro by exposing each individuals’ baseline microbiota to the same dietary fiber as used for the 12-weeks in vivo intervention. A significant correlation was shown between the in vitro and human fecal microbiota composition after 8- and 12-weeks intervention (*p* = 0.003 and *p* = 0.0107, respectively). Microbial taxa responding to the intervention in vitro and in vivo also showed clear overlap (*p* = 0.002).

**Discussion:**

These results demonstrate that in vitro models may enable pre-study selection of donors whose microbiotas respond to a specific intervention.

## Introduction

Dietary fibers are key modulators of gut microbiota composition and hold great potential for restoring intestinal homeostasis. Epidemiological studies consistently show that higher fiber intake is associated with improved health outcomes, whereas low fiber intake increases the risk of disease (Reynolds et al., 2019). However, translating these epidemiological findings into specific clinical studies with broad applicability remains challenging, given the vast diversity of dietary fiber types and the complexity of daily food intake patterns. Consequently, testing all relevant variables individually within a clinical setting is difficult.

Microbiota studies are very complex, as the human intestinal tract hosts highly diverse microbial communities composed of hundreds to thousands of bacterial, archaeal, and fungal species that coexist in complex, dynamic networks with substantial interindividual variation in composition, abundance, and function (Blaak et al., 2020). Furthermore, these communities are usually not exposed to single fiber in our daily diet, but to complex mixtures of fiber and other nutrients in highly variable amounts.

Most fibers cannot be metabolized by human enzymes; however, they serve as substrates for microorganisms in the gastrointestinal tract, primarily the colonizers of the colon. Several types of dietary fibers act as crucial substrates for specific gut microbes, which ferment them into short-chain fatty acids (SCFAs) like acetate, propionate, and butyrate (Van-Wehle and Vital, 2024; Nireeksha et al., 2025). For instance, acetate production one of the most abundant SCFA, is broadly attributed to specific members of the microbiota which also support cross-feeding interactions with other butyrate-producing microbes (Nireeksha et al., 2025). The SCFA profile and yield are strongly influenced by fiber type, molecular structure, and microbial community composition.

In the present study, we investigated the effects of a fiber mix composed of acacia gum and carrot powder on the gut microbiota. Acacia gum is a complex soluble dietary fiber derived from Acacia trees and primarily composed of galactan polymers (Ali et al., 2009). Both *in vitro* and *in vivo* studies have shown that acacia gum promotes the growth of beneficial gut bacteria such as *Bifidobacterium* and *Lactobacillus* species (Calame et al., 2008), and its fermentation has been associated with increased SCFA production *in vitro* (Alarifi et al., 2018). Carrot powder, produced from carrot peels, contains a mix of soluble and insoluble fibers. Its predominant fiber component, pectin, has been shown to stimulate SCFA production and induce microbiota changes *in vitro* (Van den Abbeele et al., 2021). Combining these two fibers could lead to a synergistic effect by promoting a broader range of beneficial gut bacteria and enhancing short-chain fatty acid production though this has yet to be confirmed through testing.

Clinical studies investigating dietary fiber supplementation in humans often yield inconclusive results (Rodriguez et al., 2024). While many studies report increases in specific bacterial taxa following fiber treatment, overall bacterial diversity is affected in only a few cases, and fecal SCFA concentrations generally remain unchanged (Vinelli et al., 2022). A recent meta-analysis demonstrated that only 1.5% of the compositional variation in the gut microbiota could be explained by fiber consumption, whereas 82% was attributed to inter-individual differences (Rodriguez et al., 2024). These findings underscore the complexity of microbiota studies and the need for more predictive and standardized tools.

Studying microbiota modulation in humans is a complex process. The development of gut-mimicking systems, such as the TIM-1 (Venema, 2015) and SHIME models (Zhu et al., 2024), enabled *in vitro* culturing of colonic microbial populations derived from fecal material, although these systems are limited in throughput. The introduction of higher-throughput platforms, such as the i-screen, has further advanced the evaluation of antibiotics and various prebiotic fibers on microbiota composition and metabolic activity (Fehlbaum et al., 2018; Schuren et al., 2019; Ladirat et al., 2013; Ladirat et al., 2014). The i-screen platform is a multi-well colonic *in vitro* fermentation model that uses a specialized medium designed to mimic the colonic environment. It allows for controlled studies of dietary fiber metabolism and the effects of other compounds by using fecal microbiota from human donors (Schuren et al., 2019).

Such platforms also enable the inclusion of multiple individual microbiota donors, allowing the study of personalized variations in intervention effects (Agamennone et al., 2023; Cantu-Jungles et al., 2025). Although these *in vitro* systems aim to mimic responses observed in humans, reliably assessing their translational value remains challenging due to the difficulty of making direct comparisons. Previous attempts to relate microbiota changes observed *in vitro* to those occurring *in vivo* have been limited to small case studies and typically relied on unpaired donor material (Rudzka et al., 2025; Van den Abbeele et al., 2023b; Van den Abbeele et al., 2023a). In the present study, changes in microbiota composition from the same participants of a previously published clinical trial (Eveleens Maarse et al., 2024), were correlated with outcomes measured by the i-screen platform, using fecal microbiota samples from all the 54 individuals of the total cohort.

This study builds on a recently conducted human dietary fiber intervention trial that investigated the effects of fiber supplementation on gut microbiota composition *in vivo* (Eveleens Maarse et al., 2024). Baseline fecal samples from the same participants were used to assess fiber-induced changes *in vitro* using the i-screen platform. Here, we directly compare microbiota composition changes observed *in vitro* and *in vivo* to evaluate the translational potential of the i-screen model. In addition, we examined the impact of fiber intervention on SCFA production. To our knowledge, this is the first study to correlate *in vitro* and *in vivo* microbiota composition responses within the same cohort.

## Materials and methods

### Clinical study

**Figure 1:**
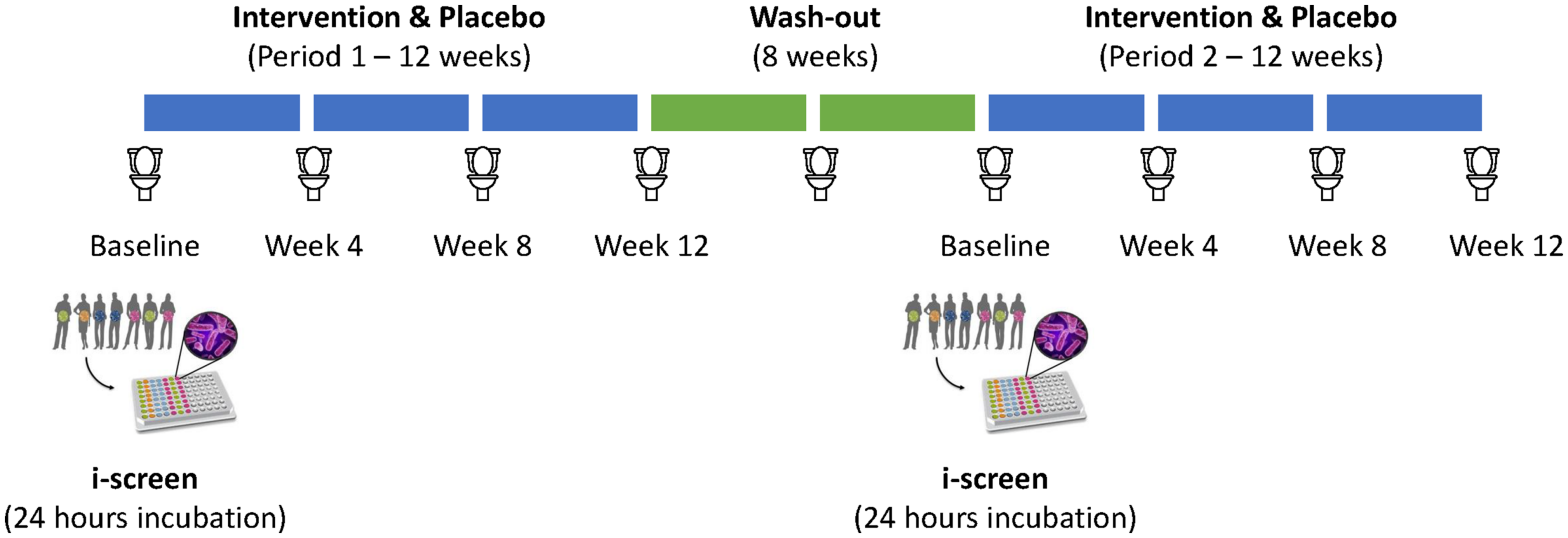
Schematic overview of the study design. Participants received either the fiber mix or a placebo for 12 weeks (Period 1), followed by an 8-week washout period. Afterwards, participants received the opposite product in a cross-over design. An *in vitro* i-screen fermentation experiment was performed using baseline fecal samples from the participants, which were incubated with the fiber mix as well as the individual fiber compounds for 24 hours.

This study builds upon the clinical research previously published by Eveleens Maarse et al. (2024) and Hogenelst et al. (2025). Briefly, we conducted a double-blind, randomized, placebo-controlled crossover study, featuring two 12-week intervention periods separated by an 8-week washout period (Figure 1).

A total of 65 healthy participants were enrolled in the study. To maximize the potential effect of the intervention, individuals with increased metabolic risk were selected, defined by a body mass index (BMI) between 25 and 30 kg/m^2^, an age range of 45–70 years, and an average daily dietary fiber intake below 30 g (Eveleens Maarse et al., 2024). All participants were required to have no clinically significant abnormalities during screening. Key exclusion criteria included the use of antibiotics, antacids, laxatives, statins, antidiarrheal, immunomodulatory, or antidiabetic medication within 3 months prior to inclusion, as well as the use of concomitant medication, vitamins, or dietary supplements within 7 days before or during the study, except for paracetamol and ibuprofen. Of the 65 enrolled participants, two were excluded for antibiotic use, one for statin use, two due to serious adverse events (SAEs), three withdrew, and three were non-compliant with the study protocol, resulting in 54 participants included in the final analysis.

All participants provided written informed consent in accordance with the Declaration of Helsinki. The study protocol received approval from the Independent Ethics Committee of the Foundation for the Evaluation of Ethics in Biomedical Research, Assen, The Netherlands. The study adhered to Good Clinical Practice (GCP) guidelines and was registered in the Toetsingonline Registry (number NL71723.056.19) and the clinicaltrials.gov registry (number NCT04829396).

### Treatment

The treatments in this crossover study with 54 healthy volunteers were the same as previously reported (Eveleens Maarse et al., 2024; Hogenelst et al., 2025). Participants consumed 13 g of powder (fiber mixture or placebo) mixed into a liquid daily for 12 weeks. The dietary fiber mixture was formulated to ensure each dose contained 10 g of Acacia Gum (AG) powder (Type 4,880, A. seyal, Willy Benecke, Germany) and 3 g of milled carrot powder (KaroPRO 1–26 SG, Food Solutions Team B. V., The Netherlands). The blend contained 10 grams of fiber per 13 grams, representing an increase to recommended levels of fiber consumption relative to the participants’ mean daily fiber intake (18.7 ± 5.9 g/day) (Eveleens Maarse et al., 2024). The placebo powder contained solely of brown (Glucidex® 19) and white (Glucidex® 17) maltodextrin (Roquette, France).

Participants were excluded if they had consumed dietary supplements (including fibers) within 7 days prior to the start of the study or during the study period, to prevent potential interference from other fiber treatments. They were instructed to maintain a stable diet throughout the study and to refrain from introducing new eating habits or dietary regimens. Diet maintenance was tracked using the Dutch Healthy Diet Index (DHDI), which remained unchanged during the study (Eveleens Maarse et al., 2024; de Rijk et al., 2021). Compliance was assessed by collecting the empty fiber supplement or placebo jars at the end of each intervention period. The jars were weighed to calculate total intake, and participants who consumed at least 80% of the assigned doses were considered compliant. Adherence to study restrictions (e.g., avoidance of concomitant medication) and the occurrence of adverse events were monitored at each study visit and through regular phone calls during the intervention periods.

### Anaerobic fermentation of fibers

Details on the i-screen model of TNO have been published (Schuren et al., 2019). Here, we used the platform with minor modifications. In brief, fecal material was collected in eSWAB tubes before each intervention period and was frozen until further use. First, the fecal samples were pre-cultured overnight. Those cultures were 50x diluted in modified standard ileal efflux medium (SIEM) to have approximately a 10^9^ CFU/mL starting concentration. All was incubated in anaerobic conditions, at 37 °C, and with shaking at 300 rpm (Ladirat et al., 2013). The medium consisted of 4.5 g NaCl, 2.5 g K_2_HPO_4_, 0.45 g CaCl_2_·2H_2_O, 0.4 g MgSO_4_·7H_2_O, 0.01 g FeSO_4_·7H_2_O, 0.4 g ox bile, 0.01 g hemin, 0.05 g pectin, 0.05 g xylan, 0.05 g arabinogalactan, 0.05 g amylopectin, 0.4 g starch, 24 g bactopeptone, 24 g casein, and 0.8 mL of vitamin mixture per liter (Wiese et al., 2022). All components were purchased from Tritium Microbiology (Veldhoven, the Netherlands). Two solutions, namely 1 M MES buffer (Sigma Aldrich) and MOPS buffer (Sigma Aldrich) were used as pH buffers. All experiments were performed in microtiter plates. The fiber mix was added at a concentration of 4 mg/mL, a dose that has shown clear microbiota-modulating effects and is within the range expected in the human gut (Ladirat et al., 2013; Agamennone et al., 2023). Single fibers, namely acacia gum and KaroPro, were added at doses of 3.07 mg/mL and 0.92 mg/mL, respectively. These concentrations were chosen to apply to the same ratio as in the clinical study (10 g and 3 g, respectively). All fibers were tested in triplicate. After 24 h of anaerobic fermentation, samples were taken for DNA isolation and SCFAs analysis.

### gDNA extraction

Genomic DNA was isolated from *in vitro* fermentation samples and human fecal material following a standard operating procedure for 96-well high-throughput extraction using zirconium bead-beating and the PurePrep 96 platform (Molgen). In brief, 150 μL of sample (maximum 250 mg fecal material) was transferred into 2.0-mL deep-well plates prefilled with 500 μL of 0.1-mm zirconium beads (BioSpec, USA). To each well, 800 μL CD1 lysis buffer (DNeasy 96 PowerSoil Pro QIAcube HT kit, Qiagen) was added. Each plate contained 50 μL of ZymoBIOMICS Microbial Community Standard (Zymo Research) as an internal process control. Plates were sealed and subjected to two bead-beating cycles of 2 min each (BeadBeater 96), with cooling on ice between cycles. Following mechanical lysis, plates were centrifuged at 3000 rpm for 6 min, and 350 μL of the supernatant was transferred to a new 96-well deep-well plate containing 350 μL Agowa Binding Buffer (LGC Genomics) and 10 μL Agowa magnetic beads per well. Samples were mixed and loaded onto the PurePrep 96 Nucleic Acid Purification System (Molgen) for automated magnetic-bead purification. The automated protocol included sequential washing with Agowa Wash Buffer 1 and Agowa Wash Buffer 2 (200 μL each), followed by elution in 65 μL Agowa Elution Buffer. Eluted DNA was sealed and stored at −20 °C until further processing.

### Amplicon sequencing

Fecal samples were collected at baseline and every 4 weeks during the study (Figure 1). Microbial DNA was extracted from all fecal samples as well as i-screen samples, and 16S rRNA gene sequencing was performed as previously described (Agamennone et al., 2023; Eveleens Maarse et al., 2024; Gart et al., 2018). Changes in the microbiota composition were analyzed by using 16S rDNA amplicon sequencing. The V4 hypervariable region was targeted as described by Kozich et al. (2013) using F515/R806 primers (Caporaso et al., 2011). Amplicon libraries were pooled in equimolar amounts and purified using the QIAquick Gel Extraction Kit (QIAGEN, Hilden, Germany). Quality was analyzed on a Fragment Analyzer (Advanced Analytical Technologies, Inc., Heidelberg, Germany) and sequencing was performed on the Illumina MiSeq platform (Illumina, Eindhoven, The Netherlands). Sequence analysis was performed using the DADA2 software package release 1.16 (Callahan et al., 2016) with the bacterial Silva database release 138.1.

### SCFAs analysis

SCFA analysis was performed as previously reported (Wiese et al., 2022). In brief, fecal or *in vitro* fermentation samples were stored at −80 °C until derivatization. For derivatization, samples were diluted in 75% methanol and mixed with internal standard (d3-acetic acid, d3-propionic acid, d3-butyric acid, and d9-valeric acid), 3-nitrophenylhydrazine (3-NPH), 1-ethyl-3-(3-dimethylaminopropyl)carbodiimide (EDC), and pyridine. The mixture was incubated for 30 min at room temperature (600 rpm) and subsequently neutralized with 2% formic acid in 25% methanol. Derivatized samples were stored at −80 °C until analysis.

Quantification of derivatized SCFAs was performed by liquid chromatography–mass spectrometry (LC–MS) using a high-resolution Q-Exactive Orbitrap mass spectrometer (Thermo Scientific, USA) equipped with a heated electrospray ionization (HESI) source and coupled to an Acquity H-Class UPLC system (Waters). Separation was achieved on an Acquity BEH C18 column (150 × 2.1 mm, 1.7 μm, Waters). SCFA concentrations were determined using calibration standards and an internal standard mix. The *in vitro* SCFAs concentration were not corrected.

### Statistical analysis

Statistical analysis was performed as described before (Eveleens Maarse et al., 2024), with some modification, particularly for the *in vitro* fermentation. Microbiota analysis was performed using R version 4.1.2 (R Core Team 2020) and illustrated using the ggplot2 package version 3.3 (Wickham and Wickham, 2016). Multivariate and microbiome diversity analysis were executed using the vegan package, version 2.5–7 (Oksanen et al., 2013). The multivariate model fitted by Principal Response Curve analysis was tested by permutation analysis in order to produce Type III (marginal) *p*-values for the terms included in the model (van den Brink et al., 2009) with 10^3^ permutations. For multivariate analyses, low-abundance taxa were removed using a cumulative read threshold, retaining features that together represented 95% of all classified reads across the dataset (624 taxa). For *α*-diversity analysis, all taxa were included without filtering, and Shannon diversity was calculated on the raw count table (without rarefaction) using the vegan package. Treatment effects on α-diversity were tested by ANOVA on a linear mixed effects (LME) model, with treatment and treatment∗time as independent variables. Post-hoc tests were performed using the emmeans package (Lenth et al., 2020). Differences in *β*-diversity were tested by repeated-measures permutational ANOVA. Fecal SCFA concentrations from the *in vivo* study were normalized to the bacterial 16S rRNA gene copy number to account for microbial load. SCFA levels obtained from the *in vitro* fermentations were not normalized, as equal colony-forming unit (CFU) densities were standardized across all conditions at baseline prior to incubation with the test compounds. The ‘lme4’ package was used for the generalized linear mixed models. We used the packages ‘vegan’ for nonmetric multidimensional scaling and PERMANOVA, ‘phyloseq’ for alpha-diversity, ‘CCA’ for RCCA and ‘ggplot2’ for visualization. Differential bacterial responses were analyzed using the limma–voom framework (edgeR, limma). ASV counts were log₂-transformed using voom, with donor pairing accounted for by including the subject as a blocking factor. Prior to modeling, ASVs were filtered to retain those present at ≥0.1% relative abundance in ≥20% of samples, and taxa with zero counts in both groups of a contrast were excluded from that comparison. Linear models were fitted for each ASV, and contrasts were defined to compare each fiber treatment (Fibre mix, Gum arabic, KaroPro) with the untreated control at 24h. Empirical Bayes moderation was applied, and significance was assessed using Benjamini–Hochberg FDR correction. Responders were identified based on changes in *Bifidobacterium breve* following the fiber intervention. Participants were classified as responders if the increase in *B. breve* at any follow-up timepoint was at least 0.5 standard deviations (SD) (Norman et al., 2003) of the baseline value. Differences in gut microbiota composition between responders and non-responders were assessed using permutational multivariate analysis of variance (PERMANOVA) based on Bray–Curtis dissimilarities (vegan package, adonis2) and two-sided Wilcoxon rank-sum test with Benjamini–Hochberg false discovery rate.

## Results

In this study, we simulated a dietary fiber intervention *in vitro* using fecal microbiota collected from the same participants who took part in the previously published clinical trial (Eveleens Maarse et al., 2024).

### *In vitro* microbiota profiling

**Figure 2:**
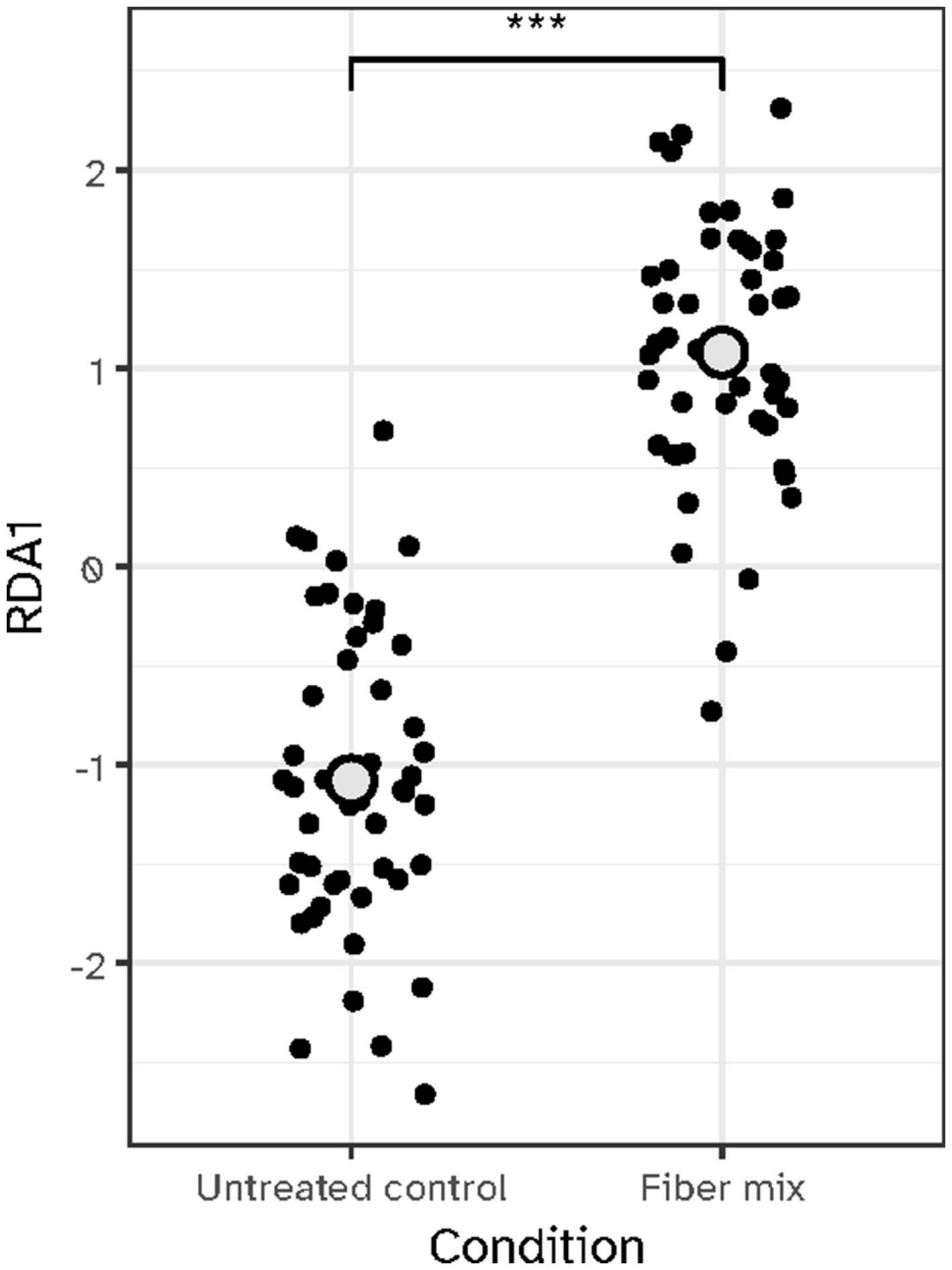
Treatment effect on beta diversity after 24-h *in vitro* exposure of individual microbiota samples to the fiber mix. A significant treatment effect (*p <* 0.001) was observed for the fiber mix compared to the untreated control, despite individual variation. Each dot represents an individual sample, and the grey circle indicates the mean for each condition. The y-axis shows RDA1, the first redundancy analysis axis.

The microbiota effects of 24-h fermentation were assessed *in vitro* by comparing the impact of the fiber mixture on each subject’s individual microbiota composition to the untreated control condition. The treatment effect was calculated using redundancy analysis (RDA), revealing a significant effect (*p* < 0.001, Figure 2). Despite the overall significant effect of the fiber mix treatment, clear individual variation was observed indicated by the wide spread of data points across the plot.

In addition to the fiber mix, the effects of the individual fibers (acacia gum and carrot powder) were also tested *in vitro*, and the results of the different exposures were analyzed using PERMANOVA. Microbial community composition differed significantly between the Fiber mix (PERMANOVA, Bray–Curtis distance; *F* = 2.96, *R*^2^ = 0.03, *p* < 0.0001) and Acacia gum (*F* = 2.47, *R*^2^ = 0.02, *p* < 0.0001) compared to the untreated control after 24 h of *in vitro* fermentation. Although the carrot powder treatment differed significantly from the untreated control at 24 h (PERMANOVA, Bray–Curtis distance; *F* = 0.44, *R*^2^ = 0.004, *p* < 0.0001), the degree of group separation was much lower than for the other treatments, potentially due to the low dose of carrot powder.

**Figure 3:**
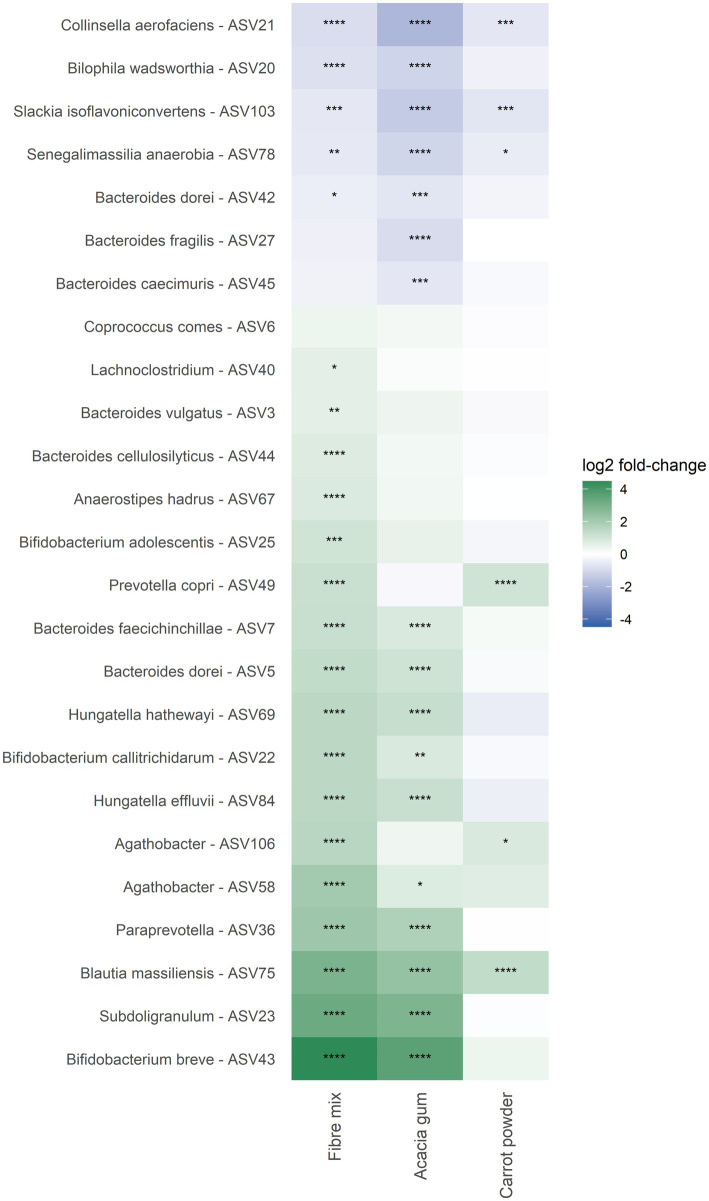
Differential bacterial responses after 24 h of *in vitro* fermentation with dietary fibers. Heatmaps show the top 25 taxa with the strongest responses to the fiber mix, selected by adjusted *p*-value (FDR) and absolute log₂ fold-change from a LIMMA analysis (paired design with subject as blocking factor). Prior to analysis, ASVs were filtered to retain taxa present at ≥0.1% relative abundance in ≥20% of samples. Shown are the corresponding log₂ fold-changes for each fiber treatment compared with the untreated control after 24 h. Rows represent bacterial taxa ordered by the fiber-mix response. Positive values (green) indicate an increase in relative abundance relative to the untreated control, while negative values (blue) indicate a decrease. Asterisks denote significant differences after multiple-testing correction (* FDR < 0.05; ** FDR < 0.01; *** FDR < 0.001; **** FDR < 0.0001).

Next, differential bacterial abundances after 24 h of *in vitro* fermentation were analyzed using the limma–voom framework, comparing each fiber treatment with the untreated control (Figure 3 and Supplementary Table S2). Clear differences in microbial composition were observed for each treatment, resulting in distinct fingerprint profiles. *Bifidobacterium breve* (untreated control, 0.22%) was strongly stimulated by the fiber mix (0.98%, *p* < 0.0001) and acacia gum (0.75%, *p* < 0.0001), whereas carrot powder (0.25%, *p* = 0.894) had no effect on this bacterium. Several *Lachnospira* species were stimulated by the fiber mix and carrot powder but not by acacia gum, suggesting additive effects when combining different dietary fibers. For example, ASV88 (untreated control, 0.00%) was stimulated by the fiber mix (0.26%, *p* < 0.0001) and carrot powder (0.29%, *p* < 0.0001), whereas acacia gum (0.00%, *p* = 0.368) had no effect. These results indicate that each individual fiber stimulates specific bacteria, while the fiber mix combines the effects of the individual components.

**Figure 4:**
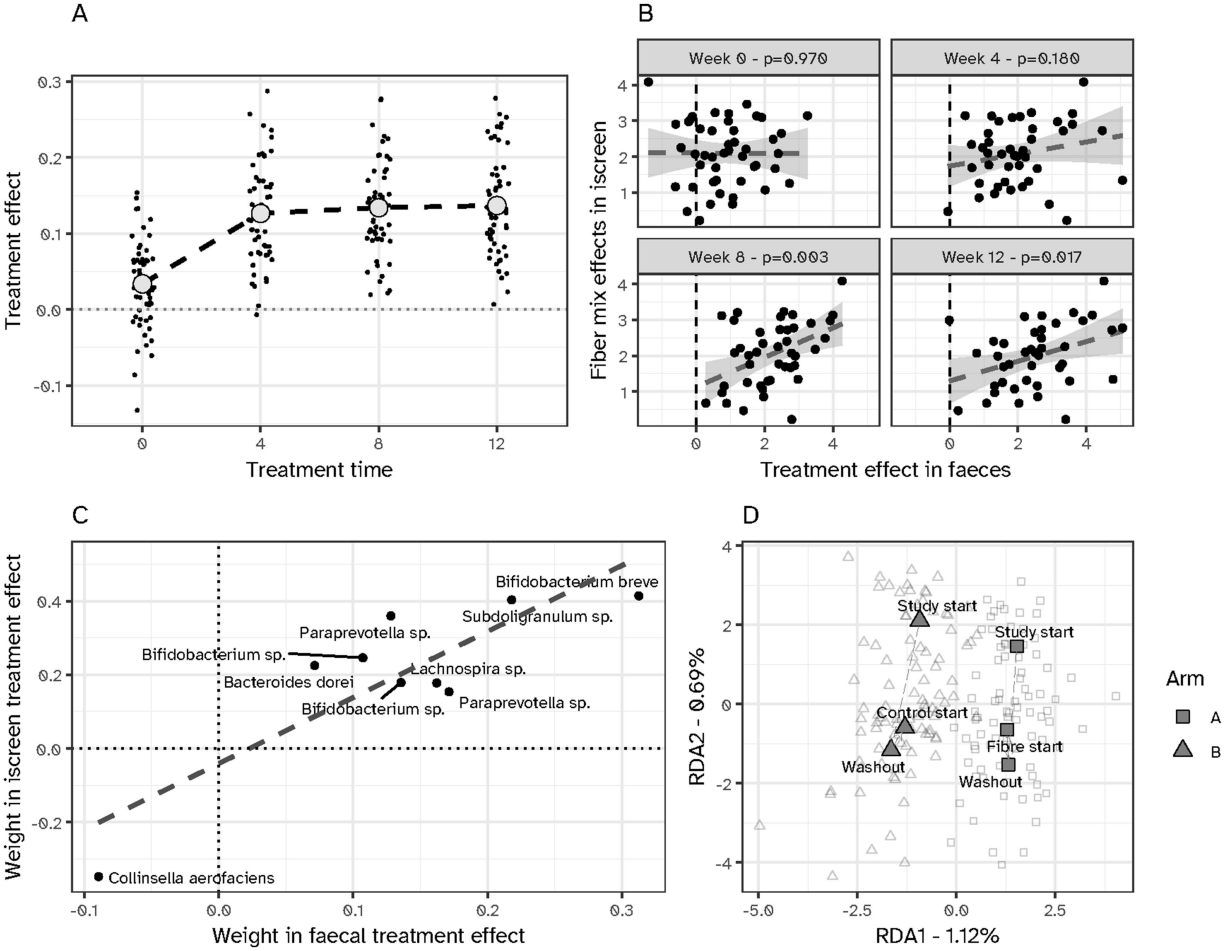
Association of the fiber mix treatment effect after *in vitro* and *in vivo* interventions. **(A)** Effect of the fiber mix intervention on *β*-diversity over time as observed in the human intervention study (Eveleens Maarse et al., 2024). The fiber intervention had a significant effect compared to the baseline (*p* < 0.001). **(B)** The *in vivo* and *in vitro* treatment effects show a significant association after 8 weeks (*p* = 0.003) and 12 weeks (*p* = 0.017) of the intervention study. **(C)** Significant overlap was observed between treatment-responsive taxa in the *in vitro* and *in vivo* results (*p* < 0.05). **(D)** Redundancy analysis (RDA) of microbiota composition at study start, start of the fiber intervention, and during the washout period. Samples from both study arms (■ Arm A with placebo/fibre; ▲ Arm B with fibre/placebo) show substantial overlap across timepoints, and no significant arm × time interaction was detected. Washout lasted 8 weeks, with an interim sample collected at 4 weeks.

In the human intervention study, an increase in beta-diversity was observed after 4 weeks of treatment (Eveleens Maarse et al., 2024). This was followed by a stable effect on beta-diversity with continued fiber consumption (Figure 4A). Next, we investigate whether the *in vivo* findings correlate with the *in vitro* results. No significant correlation in microbiota composition was observed at baseline when comparing both results (Figure 4B, *p* = 0.97). After 4 weeks of intervention, no significant correlation was observed between the *in vitro* and *in vivo* treatment effects, despite an emerging trend (*p* = 0.18). From week 8 onwards, a significant correlation was observed between the *in vitro* and *in vivo* treatment effect at week 8 (*p* = 0.03) and week 12 (*p* = 0.017). In addition to investigating microbial composition, we examined the overlap in microbial taxa responding to the interventions. A clear and significant overlap in differentially abundant taxa was observed between the two data sets (*p* = 0.002, Figure 4C). Taxa showing high increase in abundance were *Bifidobacterium breve* and *Subdoligranulum* sp., while *Collinsella aerofaciens* showed a decreased abundance. Microbial shifts observed after 24 h of *in vitro* exposure to the fiber product were comparable to those seen in the clinical study after 8 to 12 weeks of intervention.

Washout periods are common practice in clinical trials to ensure that residual treatment effects do not blunt the placebo. In the design of the human intervention study, an 8-week washout period was included, with a fecal sampling point halfway through (after 4 weeks) (Figure 1). Those samples were included in the analysis to confirm the complete washout of any potential intervention effect from the preceding treatment period. No statistically significant arm:time interaction was observed, indicating no carry-over fiber effect at the start of the second phase of the study after the 8-week washout period (Figure 4D).

Lastly, we aimed to identify responders to the fiber treatment (Kok et al., 2023). *Bifidobacterium breve* was consistently associated with the treatment in both *in vivo* and *in vitro* settings, showing a strong correlation across both formats (Figure 4C). Responders were defined as participants who demonstrated an increase in *B. breve* abundance of at least 0.5 SD from baseline (Norman et al., 2003) at one or more follow-up timepoints. Using this criterion, we identified 46 responders, whose baseline fecal microbiota composition differed significantly from the 8 non-responders (PERMANOVA, Bray–Curtis; *R*^2^ = 0.06, *F* = 3.42, *p* = 0.001). We found 55 ASVs that were significantly different at baseline between responders and non-responders (FDR corrected *p* < 0.05), including *B. breve, Bacteroides eggerthii, and Faecalibacterium prausnitzii* highlighting additional strains that may be suitable for screening purposes. Applying the same criteria *in vitro*, 31 responders and 23 non-responders were identified. However, the baseline *in vitro* composition was not statistically significant (PERMANOVA, Bray–Curtis; *R*^2^ = 0.01, *F* = 0.61, *p* = 0.74), potentially due to the pre culture that equalizes donor microbiotas. To enhance the likelihood of treatment success, pre-screening for *B. breve* using qPCR could be employed to identify potential responders, at least at *in vivo* conditions.

### Short chain fatty acid production is enhanced by mixture of fibers

**Figure 5:**
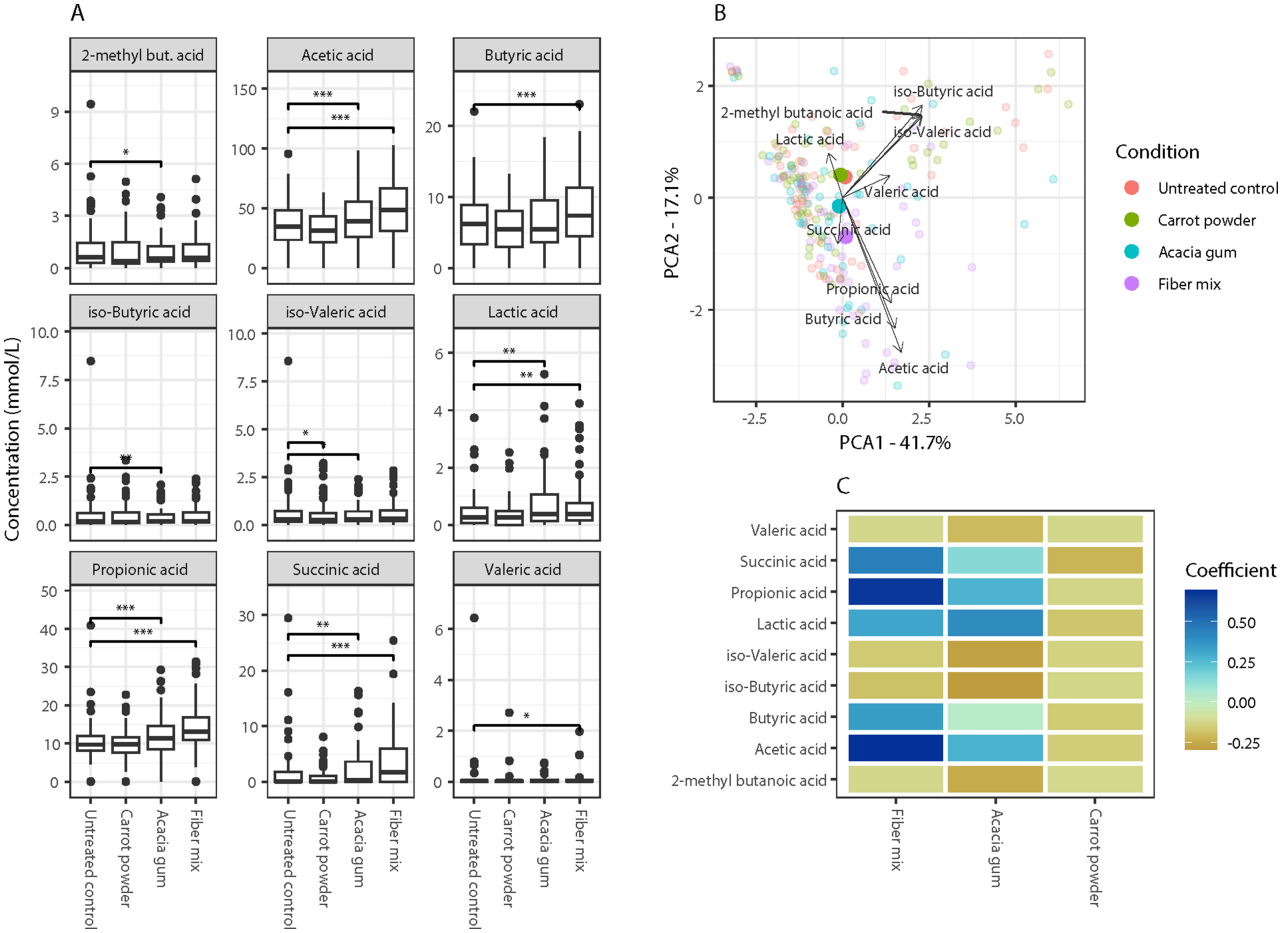
Changes in short chain fatty acids (SCFAs) after 24-h *in vitro* intervention across three treatments. **(A)** SCFAs production is in response to 24 h fermentation (untreated control, carrot powder, acacia gum and fiber mixture). Significant differences are indicated by *p < 0.05, **p < 0.01, ***p < 0.001. **(B)** Principal component analysis (PCA) plot shows correlations between SCFA concentration and the interventions; untreated control (orange, *n* = 54), carrot powder (green, *n* = 54), acacia gum (blue, *n* = 54), fiber mixture (purple, *n* = 54). The dots represent individual samples. Samples in the direction of the arrows indicate higher production of SCFAs, as shown by the respective arrows. The length of an arrow indicates the relative concentration of the SCFA. **(C)** Heatmap shows coefficients from a permutational multivariate analysis of variance (PERMANOVA) evaluating the impact of different dietary fiber interventions on SCFAs concentrations. Each tile represents the magnitude and direction of the association between a given treatment and SCFA, with color indicating the coefficient value from the PERMANOVA model. Positive coefficients indicate increases in SCFA levels under the given condition relative to the control, while negative coefficients indicate decreases.

Next, we analyzed the SCFAs produced by the microbiota metabolizing the supplemented fibers *in vitro*. In this format, we were able to compare the effects of the single fibers and the fiber mix. Overall, the fiber mix showed the strongest induction of SCFAs (Figure 5A and Supplementary Table S1), with significant effects on the production of acetic acid (*p* < 0.001), butyric acid (*p* < 0.001), propionic acid (*p* < 0.001), succinic acid (*p* < 0.001), valeric acid (*p* < 0.05) and lactic acid (*p* < 0.05). Acacia gum significantly stimulated the production of acetic acid (*p* < 0.001), propionic acid (*p* < 0.001), succinic acid (*p* < 0.01), and lactic acid (*p* < 0.01), while decreasing the production of 2-methyl butanoic acid (*p* < 0.05) and isovaleric acid (*p* < 0.05). In contrast, carrot powder only significantly stimulated the production of isovaleric acid (*p* < 0.05). This limited effect of carrot powder may be due to the low concentration used to mimic the 10:3 ratio of Acacia gum to carrot powder in the fiber mix.

The combined effects of the different interventions on SCFA production, based on principal component analysis (PCA), indicate that the effect of carrot powder is very limited (nearly identical to the untreated control), whereas the effect of acacia gum is closer to that of the fiber mix (Figure 5B). PERMANOVA analysis further supports this observation, showing a closer resemblance between Acacia gum and the fiber mix compared to carrot powder (Figure 5C). This analysis also suggests that, although carrot powder is not active on its own, in combination with Acacia gum, it leads to increased production of acetic, propionic, and butyric acids.

Next, we compared the SCFA profiles obtained *in vitro* with those measured *in vivo*. In the clinical study, no consistent changes in fecal SCFA concentrations were observed when compared with the placebo control (Eveleens Maarse et al., 2024). However, when directly compared with each participant’s baseline, several SCFAs were significantly increased in the fiber-treated group, including the branched chain fatty acids iso-butyric acid (*p* < 0.05), iso-valeric acid (*p* < 0.05), and 2-methyl butanoic acid (*p* < 0.01) (Supplementary Figure S1A). This effect was most pronounced at week 4, attenuated at week 8, and no longer statistically significant after 12 weeks of intervention. We then correlated the concentrations of each SCFA produced *in vitro* (after 24 h of fermentation) with those measured *in vivo* at different time points (4, 8, and 12 weeks) (Supplementary Figure S1B). None of the SCFAs showed a significant correlation, except for valeric acid at weeks 4 (*p* < 0.001) and 12 (*p* < 0.001). Since the i-screen operates under static conditions, accumulation of SCFAs occurs, which is not the case *in vivo* and may explain the lack of correlation between both systems.

## Discussion

In this study, we used a high-throughput fermentation system to analyze the *in vitro* effects of a fiber mixture and compared these results to the responses observed after daily consumption of the fiber mixture over 12 weeks in 54 participants. Our *in vitro* results, observed after 24 h of incubation, demonstrated a statistically significant microbiota modulation effect that aligns with the effects seen at the 8- and 12-week timepoints in the human *in vivo* intervention. These findings suggest that microbial responses to the fiber intervention observed in the *in vitro* setting are comparable to those seen in the human interventions after 12 weeks of daily fiber intake (Horvath et al., 2024; Kok et al., 2023). The *in vitro* study also showed a significant stimulation of SCFAs, with each component in the fiber mix having a differential effect.

The selection of ingredients for clinical studies is a complex process, particularly when formulating mixtures containing multiple components. High-throughput *in vitro* systems facilitate the testing of individual ingredients as well as combinations in a more efficient manner and can incorporate samples from multiple microbiome donors. However, the translatability of such *in vitro* findings to human outcomes is often debated. In this study, we replicated the human dietary fiber intervention by exposing baseline microbiota samples from each participant to a single-dose *in vitro* treatment. A strong overlap was observed between the short-term *in vitro* responses and the longer-term *in vivo* effects.

Although we demonstrate that a 24-h *in vitro* incubation significantly correlates with changes observed *in vivo* at 8 and 12 weeks, we can only speculate on why these changes occur more rapidly *in vitro*. *In vitro* systems offer a highly controlled environment where the fiber substrate is presented in a pure, undiluted form, free from the complexity as well as variability of a full dietary matrix and without any host feedback mechanisms (e.g., rapid update of SCFAs via epithelial cells). This setup allows direct microbial interaction with the fiber, leading to rapid and measurable shifts in microbiota composition and metabolic activity. In contrast, *in vivo*, the fiber is consumed as part of a complex diet, where it may be diluted or its effects modulated by other dietary components. The clean exposure *in vitro* may therefore amplify microbial responses, serving as a sensitive early indicator of potential *in vivo* outcomes. Future research should aim to elucidate the mechanisms underlying this discrepancy and to evaluate whether the i-screen can reliably predict *in vivo* microbiome responses. The present study was not designed to assess predictive capability, as the current analysis is purely correlational.

When comparing the translatability of the i-screen technology to other models, several differences become apparent. The TIM model, an *in vitro* gastrointestinal system that accurately simulates human digestive conditions, has been validated against clinical data for both nutrients and drugs (Havenaar and Bellmann, 2022). However, due to its low throughput, it is not well-suited for efficiently testing a wide range of conditions, such as varying compound doses, individual-specific responses, or multiple controls. Similarly, the SHIME model—another comprehensive gut simulation system validated for human relevance—also suffers from low throughput (Zhu et al., 2024). Moreover, these models, including the i-screen, lack a feedback mechanism to the host, highlighting the need for more complex systems that integrate human cells or tissues alongside microbiota models. Despite these limitations, the i-screen and other miniature microbiota models offer a key advantage: the ability to test multiple conditions simultaneously, making them valuable tools for high-throughput screening (Jin et al., 2022).

The presence of key species in the *in vitro* setting supports the translatability of the *in vitro* approach and enables stratification of participants prior to human intervention studies. Due to large individual variations in microbiota composition, outcomes of human intervention studies targeting microbiota are often hard to predict. By implementing a stratification procedure based on *in vitro* analysis of microbiota response to treatment, the design of human studies could be improved, increasing the scalability of experiments and yielding more predictable outcomes. However, further detailed testing is needed since this study identified responders by a single taxon without using a full stratification strategy.

Short-chain fatty acids production can be measured both *in vitro* and *in vivo* (in fecal samples). However, to date, dietary fiber interventions have generally shown negative or inconsistent results for fecal SCFAs, as these metabolites are rapidly absorbed in the intestine (den Besten et al., 2013). Similarly, in the clinical intervention examined in this study, no consistent or significant changes in fecal SCFA concentrations were observed *in vivo* compared with the placebo group (Eveleens Maarse et al., 2024). A recent meta-analysis reported that 26 out of 41 clinical studies found no significant increase in fecal SCFAs, suggesting that fecal SCFA levels are not a reliable readout for assessing fiber-induced modulation of the gut microbiota (Chen et al., 2021; Vinelli et al., 2022; Wilms et al., 2021). In contrast, *in vitro* systems can effectively measure SCFA production, as these metabolites accumulate—at least under static incubation conditions.

Our study shows that fiber intervention increases SCFAs *in vitro*, which is consistent with other studies (Harris et al., 2021). For example, various commonly used dietary fibers, such as inulin, pectin, and guar gum, are rapidly metabolized into SCFAs *in vitro*, leading to increased concentrations (Vinelli et al., 2022). These findings suggest that supplemented fibers are indeed metabolized by the colonic microbiota but are rapidly absorbed by intestinal cells and cross feeding leaving only 5–10% in the fecal samples (Quinn-Bohmann et al., 2024; Quinn-Bohmann et al., 2024). Microbiota models, like the i-screen, usually lack this absorptive capacity, leading to accumulated SCFAs levels in the system. Sampling directly from the intestine might provide better mechanistic insights, which could be more comparable to the *in vitro* setting (colonic fermentation) (van der Schoot et al., 2022). This may account for the lack of correlation between *in vivo* and *in vitro* data.

We acknowledge several limitations. For example, the present clinical study was not powered to translate findings from *in vitro* to *in vivo*, but rather served as an exploratory investigation. Future studies should account for population heterogeneity and be adequately powered to validate the translation of *in vitro* findings to *in vivo* outcomes. Additionally, refining the i-screen model to better replicate the absorptive capacity of the intestinal environment could improve correlations between *in vitro* and *in vivo* SCFA levels, which were not statistically significant in this study.

Future research should aim to establish a direct link between microbiota shifts, functional profiling, and clinical outcomes. Incorporating host metabolic, immunological, and symptomatic data in future trials would be valuable for assessing the functional relevance of observed microbial changes. For example, if a clinical study were to demonstrate an increase in specific bacteria such as *B. breve* following dietary fiber supplementation, accompanied by changes in functional and clinical outcomes (e.g., metabolic markers), and if the same pattern is observed *in vitro*, this could potentially serve as a predictor of clinical response. However, this remains speculative and requires further validation. In addition, to better understand the mechanisms underlying interindividual variability, potential contributing factors, such as baseline microbiota composition, diet, and lifestyle, should be explored in greater depth. This requires the selection of a culture medium that adequately represents the individual variability in participants’ diets. In the present study, a standard SIEM medium was used, formulated to mimic human ileal efflux; however, it may not fully account for differences in dietary composition among participants.

In summary, human intervention studies concerning the complexity of gut microbiota are challenging and time-consuming (requiring a minimum of 8 weeks of intervention for modulation), while an *in vitro* fermentation system can reduce variables and perform in high throughput within shorter timeframes (here, 24 h). Additionally, one-size-fits-all approaches usually fail due to individual responses, particularly in complex microbiota systems (Kok et al., 2023). The *in vitro* platform could be used to screen individuals that potentially respond to fiber treatment; however, this is still to be tested. The proposed approach for *in vitro* pre-screening of fecal material opens the way to improve the efficacy and possibility of individual microbiota-tailored interventions. Therefore, 24 h of incubation in the i-screen platform can serve as a high-throughput initial investigation, offering early indications of microbiota-driven responses and providing useful clues to guide further studies.

## Data Availability

The data generated and analyzed in this study have been deposited in the European Nucleotide Archive (ENA) under the accession number PRJEB81519. This data is publicly available and can be accessed at ENA.
