# Supplementary material for: Translating in vitro gut microbiota models to human context: compositional correlations under dietary fiber intervention
Source: Front Microbiol. 2025 Dec 18;16:1708906. doi: 10.3389/fmicb.2025.1708906 (PMC12756460; doi:10.3389/fmicb.2025.1708906)
Supplement: Supplementary file 1 [file Data_Sheet_1.docx]

**Supplementary Tables and Figures**

**
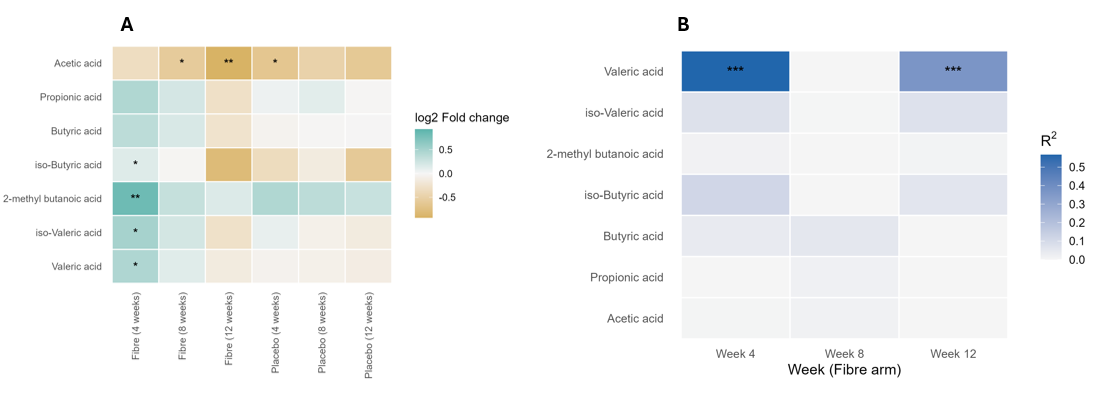
**

**Figure S1.** (A) Heatmap of adjusted fecal SCFAs (concentrations adjusted for log(16S)) showing mean log₂ fold change vs baseline (week 0) within the same treatment arm at weeks 4, 8, and 12. Positive tiles indicate increases, negative tiles decreases. Asterisks indicate FDR-corrected significance vs baseline (paired Wilcoxon). (B) Like-with-like correlations between *in vitro* i-screen (24 h, fibre mix; raw SCFAs) and *in vivo* adjusted fecal SCFAs at weeks 4, 8, and 12. Tiles show Pearson R² across subjects; asterisks denote FDR-adjusted significance (* FDR<0.05, ** FDR<0.01, *** FDR<0.001).

**Supplementary Table S1:** Overview of short-chain fatty acid (SCFA in mM) concentrations measured in the *in vitro* i-screen fermentation model after 24h of incubation with different dietary fibre substrates. Each value represents the mean concentration across all subjects (averaged per subject from technical replicates). Statistical comparisons were performed against the untreated control (24h) using paired Wilcoxon signed-rank tests (subjects as pairs). Benjamini–Hochberg false discovery rate (FDR)–adjusted p-values.

| **SCFAs** | **Untreated control (24h)** | **Fibre mix (24h)** | **Acacia gum (24h)** | **Carrot powder (24h)** | **FDR (Fibre mix)** | **FDR (Acacia gum)** | **FDR (Carrot powder)** |
| --- | --- | --- | --- | --- | --- | --- | --- |
| Acetic acid | 33,02 | 49,72 | 42,59 | 32,98 | 2,76E-08 | 1,07E-06 | 8,17E-01 |
| Propionic acid | 10,69 | 14,99 | 12,48 | 9,78 | 1,61E-06 | 8,10E-04 | 1,00E+00 |
| Butyric acid | 6,63 | 8,17 | 6,74 | 5,81 | 6,06E-06 | 7,90E-02 | 4,40E-01 |
| iso-Butyric acid | 0,77 | 0,64 | 0,50 | 0,60 | 9,19E-01 | 1,86E-01 | 1,56E-01 |
| 2-methyl butanoic acid | 1,33 | 1,20 | 1,02 | 1,14 | 6,56E-01 | 4,91E-01 | 1,50E-01 |
| iso-Valeric acid | 0,87 | 0,74 | 0,61 | 0,69 | 6,56E-01 | 4,91E-01 | 1,00E-01 |
| Valeric acid | 0,31 | 0,28 | 0,21 | 0,21 | 7,52E-05 | 9,65E-02 | 1,00E-01 |
| Lactic acid | 0,50 | 0,77 | 0,84 | 0,38 | 3,54E-02 | 2,37E-01 | 2,12E-03 |
| Succinic acid | 1,91 | 3,79 | 2,60 | 1,05 | 2,88E-05 | 9,65E-02 | 8,17E-01 |

**Supplementary Table S2:** Differentially abundant taxa after 24h *in vitro* fermentation with dietary fibres.

The table lists the 25 taxa displayed in Figure 3, selected based on the limma–voom analysis. Columns show the mean relative abundance (%) of each ASV in the untreated control, Fibre mix, Acacia gum, and Carrot powder incubations (all at 24h), the taxonomic label as displayed in the heatmap, and the corresponding false-discovery-rate-adjusted p-values (FDR) for each fibre treatment versus the untreated control.

| **ASV** | **Fibre mix (24h)** | **Acacia gum (24h)** | **Carrot powder (24h)** | **Untreated control (24h)** | **Taxon label** | **FDR (Fibre mix)** | **FDR (Acacia gum)** | **FDR (Carrot powder)** |
| --- | --- | --- | --- | --- | --- | --- | --- | --- |
| ASV43 | 1,42 | 1,11 | 0,48 | 0,38 | Bifidobacterium breve - ASV43 | 7,11E-25 | 1,26E-17 | 4,56E-01 |
| ASV23 | 1,70 | 1,88 | 0,09 | 0,11 | Subdoligranulum - ASV23 | 1,02E-14 | 4,73E-12 | 9,37E-01 |
| ASV75 | 0,69 | 0,74 | 0,55 | 0,44 | Blautia massiliensis - ASV75 | 1,09E-20 | 9,37E-15 | 1,81E-05 |
| ASV36 | 1,23 | 1,32 | 0,06 | 0,04 | Paraprevotella - ASV36 | 1,98E-12 | 4,74E-09 | 9,60E-01 |
| ASV58 | 0,68 | 1,00 | 0,61 | 0,52 | Agathobacter - ASV58 | 3,39E-10 | 2,26E-02 | 1,05E-01 |
| ASV106 | 0,28 | 0,29 | 0,21 | 0,16 | Agathobacter - ASV106 | 2,95E-07 | 2,40E-01 | 1,83E-02 |
| ASV84 | 0,33 | 0,35 | 0,04 | 0,04 | Hungatella effluvii - ASV84 | 1,99E-08 | 5,53E-06 | 1,98E-01 |
| ASV22 | 1,39 | 1,25 | 0,27 | 0,21 | Bifidobacterium callitrichidarum - ASV22 | 9,77E-07 | 5,52E-03 | 6,59E-01 |
| ASV69 | 0,54 | 0,63 | 0,06 | 0,07 | Hungatella hathewayi - ASV69 | 4,62E-07 | 1,86E-05 | 2,15E-01 |
| ASV5 | 5,75 | 5,93 | 2,89 | 2,87 | Bacteroides dorei - ASV5 | 3,27E-09 | 2,13E-06 | 6,59E-01 |
| ASV7 | 3,96 | 3,96 | 2,56 | 1,87 | Bacteroides faecichinchillae - ASV7 | 3,26E-10 | 8,63E-06 | 4,56E-01 |
| ASV49 | 0,98 | 0,25 | 1,22 | 0,22 | Prevotella copri - ASV49 | 1,80E-07 | 4,47E-01 | 3,18E-05 |
| ASV25 | 1,28 | 1,14 | 0,79 | 0,79 | Bifidobacterium adolescentis - ASV25 | 6,88E-04 | 9,75E-02 | 5,43E-01 |
| ASV67 | 0,49 | 0,50 | 0,47 | 0,47 | Anaerostipes hadrus - ASV67 | 8,73E-05 | 1,44E-01 | 9,60E-01 |
| ASV44 | 0,73 | 0,67 | 0,63 | 0,55 | Bacteroides cellulosilyticus - ASV44 | 7,37E-05 | 1,45E-01 | 7,06E-01 |
| ASV3 | 8,58 | 8,63 | 6,17 | 6,37 | Bacteroides vulgatus - ASV3 | 3,03E-03 | 8,17E-02 | 5,91E-01 |
| ASV40 | 0,72 | 0,77 | 0,76 | 0,69 | Lachnoclostridium - ASV40 | 1,34E-02 | 6,68E-01 | 9,60E-01 |
| ASV6 | 3,02 | 3,18 | 3,10 | 2,71 | Coprococcus comes - ASV6 | 1,12E-01 | 2,49E-01 | 8,08E-01 |
| ASV45 | 0,43 | 0,40 | 0,68 | 0,63 | Bacteroides caecimuris - ASV45 | 7,65E-02 | 4,39E-04 | 4,71E-01 |
| ASV27 | 0,87 | 0,69 | 1,29 | 1,07 | Bacteroides fragilis - ASV27 | 8,31E-02 | 7,62E-06 | 9,99E-01 |
| ASV42 | 0,50 | 0,51 | 0,64 | 0,73 | Bacteroides dorei - ASV42 | 2,37E-02 | 1,32E-04 | 2,15E-01 |
| ASV78 | 0,13 | 0,14 | 0,20 | 0,29 | Senegalimassilia anaerobia - ASV78 | 2,16E-03 | 4,74E-09 | 1,83E-02 |
| ASV103 | 0,10 | 0,06 | 0,11 | 0,21 | Slackia isoflavoniconvertens - ASV103 | 6,97E-04 | 1,56E-14 | 4,31E-04 |
| ASV20 | 0,87 | 0,82 | 1,32 | 1,62 | Bilophila wadsworthia - ASV20 | 3,25E-06 | 2,05E-10 | 1,23E-01 |
| ASV21 | 0,96 | 0,67 | 1,33 | 1,92 | Collinsella aerofaciens - ASV21 | 5,36E-08 | 1,97E-26 | 4,04E-04 |
